# Development of Performance, Physiological and Technical Capacities During a Six-Month Cross-Country Skiing Talent Transfer Program in Endurance Athletes

**DOI:** 10.3389/fspor.2020.00103

**Published:** 2020-08-12

**Authors:** Rune Kjøsen Talsnes, Tor-Arne Hetland, Xudan Cai, Øyvind Sandbakk

**Affiliations:** ^1^Meråker High School, Trøndelag County Council, Steinkjer, Norway; ^2^Department of Sports Science and Physical Education, Nord University, Bodø, Norway; ^3^School of Physical Education and Sport Training, Shanghai University of Sport, Shanghai, China; ^4^Olympic Games Preparation Office, Chinese Olympic Committee, Beijing, China; ^5^Department of Neuromedicine and Movement Science, Centre for Elite Sports Research, Norwegian University of Science and Technology, Trondheim, Norway

**Keywords:** aerobic capacity, cycle length, gross efficiency, endurance training, strength training, Olympics

## Abstract

**Purpose:** To examine the development of performance, physiological and technical capacities as well as the effect of sport background among runners, kayakers and rowers when transferred to cross-country (XC) skiing over a 6-month training period.

**Methods:** Twenty-four endurance athletes (15 runners and 9 rowers/kayakers; 15 men and 9 women) were tested for performance, physiological and technical capacities during treadmill running and roller-ski skating, double-poling ergometry, as well as upper-body, one-repetition maximum-strength (1 RM) at baseline (pre) after three (mid) and 6-months (post) of XC ski-specific training.

**Results:** Peak treadmill speed when roller-ski skating improved significantly (13%, *P* < 0.01) from pre-post, with a larger improvement in runners than in kayakers/rowers (16 vs. 9%, *P* < 0.05), whereas peak speed in running was unchanged. Average power output during 5-min and 30-s ergometer double-poling tests improved by 8% and 5% (both *P* < 0.01), with improvement found only in runners on the 30-s test (8 vs. −2% in kayakers/rowers, *P* < 0.01). Peak oxygen uptake (VO_2peak_) in running and double-poling ergometry did not improve, whereas VO_2peak_ in roller-ski skating improved by 5% in runners (*P* < 0.05). Submaximal gross efficiency increased by 0.6%-point and cycle length by 13%, whereas 1 RM in seated pull-down and triceps press increased by 12 and 11%, respectively (all *P* < 0.05).

**Conclusion:** Six-months of XC ski-specific training induced large improvements in sport-specific performance which were associated with better skiing efficiency, longer cycle length, and greater 1RM upper-body strength in a group of endurance athletes transferring to XC skiing. Furthermore, larger sport-specific development was found in runners compared to kayakers/rowers.

## Introduction

Cross-country (XC) skiing is a traditional Winter Olympic sport, involving combined upper-and lower-body exertion of varying intensity in competitions lasting from ~3 min (~1.3–1.8 km) in sprint races to more than 2 h (≤50 km) in the longest distance races (FIS, 2019). The constant changes in terrain leads to fluctuations in speed, work rate, and metabolic intensity as well as utilization of different sub-techniques of skating and classical styles both during training and competitions (Sandbakk et al., 2011a, 2016b; Bolger et al., 2015; Andersson et al., 2017; Karlsson et al., 2018; Haugnes et al., 2019; Losnegard, 2019). Accordingly, XC skiing demands high levels of both aerobic and anaerobic energy delivery capacities, strength and speed, as well as technical and tactical expertise (Sandbakk and Holmberg, 2017; Losnegard, 2019).

Traditionally, world-class XC skiers have been tested in running for physiological capacities and have demonstrated maximum oxygen uptake (VO_2max_) values among the highest ever reported in the scientific literature, with values of 80–90 and 70–80 mL·kg^−1^·min^−1^ reported for men and women, respectively (Saltin and Astrand, 1967; Ingjer, 1992; Tønnessen et al., 2015; Haugen et al., 2018). However, because of the technical complexity of XC skiing, sport-specific peak oxygen uptakes (VO_2peak_) above 90% of VO_2max_ combined with high fractional utilization of VO_2peak_ in all the main sub-techniques in classical and skating are additionally considered as important capacities in modern XC skiing (Sandbakk and Holmberg, 2017). In addition, sport-specific performance in the laboratory (e.g., time to exhaustion and peak speed) during treadmill roller-skiing using both skating and classical techniques has repeatedly been associated with on-snow performance (Sandbakk et al., 2011a, 2016b) and used to distinguish skiers on different performance levels (Sandbakk et al., 2011b, 2016a).

Furthermore, XC skiers must effectively convert metabolic energy into external work rate and speed, and thus, gross efficiency has been shown to be a key determinant of performance in both sprint and distance XC skiing (Sandbakk et al., 2010, 2011b, 2012, 2013; Ainegren et al., 2013). In addition, better performing skiers often demonstrate a skiing technique with longer cycle lengths and lower cycle rates, indicating better technique-specific propulsion that could possibly explain variations in work economy and skiing efficiency (Sandbakk et al., 2010; Sandbakk and Holmberg, 2017). Clearly, an adequate level of both upper- and lower-body strength and power are needed to produce and transfer these forces efficiently. Especially in sprint XC skiing, maximal strength within movement-specific exercises as well as upper-body power have previously been shown to be correlated with performance (Stöggl et al., 2007).

The training of XC skiers targets a concurrent development of the abovementioned capacities, with training programs especially designed to facilitate long-term development of aerobic energy delivery and skiing efficiency (Holmberg, 2015; Sandbakk and Holmberg, 2017). Today, world-class XC skiers perform an annual training volume of approximately 750–950 h, with 90% being endurance training with a polarized intensity distribution (Sandbakk and Holmberg, 2017), whereas 10% is performed as strength and speed training (Tønnessen et al., 2014; Sandbakk and Holmberg, 2017; Solli et al., 2017). XC skiers typically perform 50–60% of their training in sport-specific exercise modes (i.e., roller-skiing and skiing) and the reminder mainly in running during the preparation period, whereas during the competitive season most of the training is performed as sport-specific (Tønnessen et al., 2014; Sandbakk et al., 2016a; Sandbakk and Holmberg, 2017; Solli et al., 2017). These training characteristic in XC skiing are largely based on retrospective findings, and there is currently a lack of longitudinal designs following this sophisticated training puzzle and its subsequent effects on endurance and performance adaptations.

Aiming for XC skiing success at the Beijing Winter Olympic Games in 2022, China has developed a talent transfer program (also referred to as athlete transfer program) in which athletes from various summer sports (e.g., running, rowing, and kayaking) are transferred to XC skiing by utilizing state-of-the-art coaching and training methods (Sandbakk and Holmberg, 2017). Although transfer of individual athletes (Rønnestad et al., 2019) and groups (Bullock et al., 2009; MacNamara and Collins, 2015) to new sports are commonly used in sports practice, the effects of such transfer programs in endurance sports have not been documented in the scientific literature. In addition, an individual's physiological and anthropometrical potential for successful transfer to another sport and the underlying mechanisms explaining the development are currently unexplored (MacNamara and Collins, 2015). Therefore, the primary aim of this study was to examine the development of performance, physiological and technical capacities among runners and kayakers/rowers when transferred to XC skiing over a 6-month period of XC ski-specific training. A secondary aim was to examine the effect of sports background on this development. We hypothesized that a rapid development of XC ski-specific performance, physiological and technical capacities would be achieved, with less improvement in less sport-specific modes among such already well-trained endurance athletes.

## Methods

### Participants

The participants consisted of 24 young Chinese talent transfer athletes with background in various summer endurance sports. Fifteen were reported as previous runners (i.e., long and middle distance) and 9 as previous kayakers or rowers. For all analyses, kayakers (*n* = 7) and rowers (*n* = 2) were pooled. The groups consisted of 15 men and 9 women, with 4 and 5 women reported as being runners and kayakers/rowers, respectively. The athletes' sports backgrounds, ages, and anthropometrics are presented in Table 1. All athletes were selected from the group of the second-best athletes in their respective sports in China and had trained professionally for this sport for several years. Therefore, they were given the opportunity to transfer from their summer sport to XC skiing while aiming for participation in the Beijing 2022 Olympic Games (verbal communication with members of the Chinese Olympic Committee).

**Table 1:** Baseline characteristics (mean ± SD) of the 24 endurance transfer athletes participating in the study.

**Variables**		**Sport background**	
	**All pooled**	**Running**	**Kayaking/rowing**
	**(*n* = 24)**	**(*n* = 15)**	**(*n* = 9)**
Age (yrs)	19.2 ± 1.8	18.7 ± 2.0	20.0 ± 1.2
Body height (cm)	174.9 ± 10.2	173.7 ± 10.1	176.8 ± 10.1
Body mass (kg)	65.4 ± 9.9	62.1 ± 8.6	71.0 ± 9.3^*^
Body mass index (kg·m^−2^)	21.3 ± 1.6	20.5 ± 1.3	22.6 ± 8.1**

* Significant difference between runners and kayakers/rowers (**P < 0.01; *P < 0.05).

### Ethics Statement

The Regional Committee for Medical and Health Research Ethics waives the requirement for ethical approval for such studies. Therefore, the ethics of the study is done according to the institutional requirements and approval for data security and handling was obtained from the Norwegian Center for Research Data. Prior to the data collection, all participants provided written informed consent to voluntarily take part in the study. The participants were informed that they could withdraw from the study at any point in time without providing a reason for doing so.

### Overall Design of the Study

After an initial 3.9 ± 1.9-month introduction to XC roller-skiing in China, the athletes' performance, physiological and technical capacities during treadmill running, treadmill roller-ski skating, double-poling ergometry, as well as upper-body, one-repetition maximum-strength (1RM) were measured at three different timepoints during the 6-month training period. In addition, detailed descriptions of the athletes training were recorded. Pre-tests (baseline) were performed initially when the transfer program started in November 2018, followed by mid-tests in February 2019 and post-tests in May 2019. The entire battery of tests was conducted over a 5-day period, including 3 days of testing for each athlete with 2 days of easy training in between. The easy training days consisted of one session of 90-min low-intensity roller-skiing or skiing. The athletes' training loads were standardized in the last 2 days prior to test day 1 with a protocol used to optimize their fitness levels and performance (i.e., rest day followed by a short running session including 2 × 5-min stages at moderate intensity with 3-min recovery in between). During each week of testing, test day 1 consisted of physiological and performance testing during treadmill running followed by upper-body 1RM tests. Test day 2 consisted of physiological and performance testing as well as testing of cycle characteristics during treadmill roller-ski skating, whereas on test day 3 the athletes were tested for upper-body physiological capacities and performance using double-poling ergometry. Prior to all tests, the athletes completed a standardized 10-min low-intensity warm-up by running on a treadmill, being instructed to keep an exercise intensity corresponding to 3 on a 1–10-point rating of perceived exertion (RPE) scale. All test procedures, including the order of the tests and the time of day were similar for all three test timepoints. The athletes were allowed to drink sports drinks *ad libitum* during testing and were actively encouraged to drink in between the running and strength tests on test day 1.

### Training Monitoring

During the 6-month training period, all athletes followed a standardized XC ski-specific training program. However, each athlete had a personal coach who helped them to daily adjust their training to ensure optimized training load and adaptations for each individual athlete. A typical training week normally consisted of two daily training sessions (i.e., morning session at 09:00 AM and afternoon session at 04:00 PM). In addition, a third session conducted earlier in the morning (i.e., 08:00 AM) were customized and developed for the project. These sessions were named “XC skiing drills” and had a duration of approximately 30-min with focus on developing basic and fundamental skills such as balance, coordination, and stabilization using various exercises targeting muscles involved in the force transfer and functional movement of XC skiing. Day-to-day training data were registered and systematized in detail for all athletes according to the so-called “modified session-goal approach” (Sylta et al., 2014). Training recorded for each session included total training time distributed across training form, exercise mode, and intensity zone as described in detail elsewhere (Tønnessen et al., 2014; Solli et al., 2017). Distribution of endurance-training intensity was reported using a three-zone scale (LIT, low-intensity; MIT, moderate-intensity; and HIT, high-intensity) based on the ventilatory changes corresponding to the first and second lactate thresholds (Seiler and Kjerland, 2006; Solli et al., 2017). Days and/or sessions where the athletes were not able to follow standardized training due to injury and/or illness were verified by a medical doctor and registered. All training data were registered and systematized by Norwegian coaches and researchers contributing to the project.

### Instruments and Materials

Treadmill running was performed on a 2.5 × 0.7 m motor-driven treadmill (RL 2500E, Rodby, Södertalje, Sweden), whereas treadmill roller skiing was performed on a 3.5 × 2.5 m treadmill (RL 3500E, Rodby, Södertalje, Sweden). The treadmill belt consisted of non-slip rubber surface, allowing the athletes to use their own poles (pole length of 157 ± 9 cm) with special carbide tips during treadmill roller-skiing. All athletes used the same pair of skating roller-skis with standard category 2 wheels (IDT Sports, Lena, Norway) to reduce variations in rolling resistance. Before each test session, roller-skis were pre-warmed through 20-min of roller-skiing on the treadmill and rolling friction force (Ff) measured with a towing test as previously described by Sandbakk et al. (2010), providing an average μ value of 0.023, which was included in the calculation of work rate.

Cycle characteristics (i.e., cycle rate and cycle length) were determined by 2-D video recordings using an Apple iPad 4 (Apple Inc., California, USA) at 30 frames per second. The video recordings were further analyzed by the software Coaches Eye (TechSmith Corporation, Michigan, USA). The iPad was positioned on the left side of the treadmill, 90° to the athletes' skiing direction, providing a total view of the movement range of both skis and poles. Cycle time was taken as the time between two pole plants, and average cycle characteristics were determined by timing five cycles. Cycle length was then calculated by multiplying the speed of the treadmill by cycle time, whereas cycle rate was taken as the reciprocal of cycle time as described in previous studies (Sandbakk et al., 2010).

Double-poling testing was conducted using a Concept2 SkiErg (Morrisville, VT, USA) double-poling ergometer with the damper positioned at drag setting 7 to ensure adequate work rate and exercise intensity. The athletes performed all tests in an upright position wearing running shoes and were instructed to simulate double-poling on skis. In addition, the distance between the athlete and the ergometer was standardized and similar for all athletes and tests to ensure that the movements most closely simulated double-poling on skis. Power output was measured with the ergometer's internal software, and the athletes were able to see their values on the screen during testing. 1RM upper-body strength tests were performed in an Impulse cable crossover apparatus (Impulse, Midlothian, Scotland) with a customized rope used to simulate the handles of XC skiing poles.

Respiratory variables were measured using open-circuit indirect calorimetry with mixing chamber and 30-s sampling time (Oxycon Pro, Jaeger GmbH, Hoechberg, Germany). The instruments were calibrated against ambient air conditions and certified gases of known concentrations of O_2_ (15.0%) and CO_2_ (5.0%) before each test session. The flow transducer (Triple V, Erick Jaeger GmbH, Hoechberg, Germany) was calibrated using a 3-L high-precision calibration syringe (Calibration syringe D, SensorMedics, Yorba Linda, CA, USA). Heart rate was continuously measured with a Garmin Forerunner 935 (Garmin Ltd., Olathe, KS) and synchronized with the Oxycon Pro system. Blood lactate in 20 μL of blood was taken from the fingertip and measured using the stationary Biosen C-Line lactate analyser (Biosen, EKF Industrial Electronics, Magdeburg, Germany). The device was calibrated every 60 min with a 12 mmol·L^−1^ standard concentration. RPE was determined using a 1–10 point RPE scale (Haddad et al., 2017). The athletes' body masses were measured using a precise weight (Seca, model 708, GmbH, Hamburg, Germany) and body height using a calibrated stadiometer (Holtain Ltd., Crosswell, UK).

### Test Protocols and Measurements

#### Treadmill Running Tests (Test Day 1)

Physiological and performance testing in treadmill running was conducted using protocols developed by the Norwegian Top Sport Center as previously described (Ingjer, 1992; Tønnessen et al., 2015). Submaximal lactate profile testing was considered complete when the athletes reached a blood lactate value of ≥4 mmol·L^−1^. Treadmill speed at 4 mmol·L^−1^ was calculated using linear interpolation (Sjödin et al., 1982). After a 5-min recovery, the athletes conducted an incremental test to determine VO_2max_ and performance measured as peak treadmill speed (V_peak_) calculated according to Sandbakk et al. (2011b).

#### Upper-Body Strength Tests (Test Day 1)

After a 20-min recovery, the athletes were tested for 1RM upper-body strength in the ski-specific exercises, seated pull-down and triceps press, using protocols described in detail by Losnegard et al. (2011). All 1RM tests were conducted using the same equipment with identical equipment positioning for each athlete, and all tests were supervised by the same test leader who gave verbal feedback to ensure proper technique. All athletes were familiar with the exercises before testing.

#### Treadmill Roller-Ski Skating Tests (Test Day 2)

Initially, submaximal lactate profile testing was performed at a constant speed (2.5 m·s^−1^) and starting incline of 1° using a graded protocol, including 3–6 periods of 5-min stages with a stepwise increase in workload (1°) and 60-s recovery in between each stage. Heart rate was defined as the average of the last 30 s of each stage, whereas RPE and blood lactate values were determined directly after completing each stage. At a given submaximal workload (3°), VO_2_ and video recordings were included between the third and fifth minute of the stage to determine cycle characteristics as well as calculations of submaximal oxygen cost (O_2_-cost) and efficiency. Gross efficiency was used as a measure of efficiency and defined as the ratio of work rate and metabolic rate as described by Sandbakk et al. (2010). The athletes were instructed to use the skating G3 sub-technique during this stage, whereas they were instructed to freely choose sub-techniques for the rest of the test. The submaximal test was considered completed when the athlete reached a blood lactate value of ≥4 mmol·L^−1^. Power output at 4 mmol·L^−1^ was calculated using linear interpolation (Sjödin et al., 1982). After a 5-min recovery period, VO_2peak_ and performance measured V_peak_ calculated according to Sandbakk et al. (2011b) were determined. Incremental testing was used with a starting incline and speed of 4° and 2.5 m·s^−1^, respectively. The incline was kept constant, while the speed was subsequently increased by 0.28 m·s^−1^ every 60 s until voluntary exhaustion. Respiratory variables and heart rate were measured continuously, and VO_2peak_ was defined as the average of the two highest and consecutive 30-s measurements. Peak heart rate (HR_peak_) was defined as the highest 5-s heart rate measurement during the test, whereas RPE was determined directly after, and blood lactate values 1 min after, completing the test.

#### Double-Poling Ergometer Tests (Test Day 3)

Initially, the athletes performed 3-min specific warm-up (RPE = 4) on the double-poling ergometer after completing the 10-min standardized warm-up. Thereafter, all athletes conducted a modified 30-s Wingate test and a 5-min self-paced performance test with a 5-min recovery period in between using protocols similar to those in previous studies of XC skiing (Hegge et al., 2015, 2016). The athletes were instructed to perform the 30-s Wingate test as all-out, whereas based on previous training using double-poling ergometry, instructions for an even, maximal pacing were given prior to the 5-min test to prevent “overpacing.”

### Statistical Analysis

All data are reported as means ± standard deviations (SD). Assumption of normality was tested with a Shapiro-Wilk test in combination with visual inspection of the data. Baseline and training characteristics between-groups were compared using an independent samples *t*-test. A General Linear Model (GLM) repeated measures design was used to analyze changes in the different laboratory capacities, followed by Bonferroni *post-hoc* corrections to localize differences between the specific time-points. Between-group differences in delta changes were examined using a univariate GLM analysis of covariance (ANCOVA) adjusted for baseline (pre) values. Furthermore, effect sizes (ES) were calculated according to Cohens d, and interpretations of the magnitude were as follows: 0–0.2 = trivial, 0.2–0.6 = small, 0.6–1.2 = moderate, 1.2–2.0 = large, and >2 = very large (Hopkins et al., 2009). For all comparisons, statistical significance was set at an alpha level of *P* < 0.05, and alpha levels of P 0.05–0.1 were considered as trends. All data analyses were conducted using SPSS 26.0 (SPSS Inc., Chicago, IL, United States) and Excel 2016 (Microsoft Corporation, Redmond, Washington, United States).

## Results

### Training and Baseline Characteristics

Detailed descriptions of the athletes' training during the 6-month period are presented in Table 2. Runners and kayakers/rowers did not differ in the number of rest days during the 6-month training period, but runners showed an overall higher training compliance due to fewer days of injury and illness in comparison to kayakers/rowers (6 ± 2 vs. 12 ± 5 days, P = 0.08). Hence, runners completed more sessions (307 ± 15 vs. 280 ± 31, *P* < 0.05) and showed a tendency toward a higher training volume performed (358 ± 11 vs. 341 ± 21 h, *P* = 0.09) compared to kayakers/rowers. Kayakers/rowers demonstrated 12.5 and 9.3% higher body mass and body mass index (BMI) compared to runners at baseline (both *P* < 0.05; Table 1), whereas no significant changes in body mass or body height was observed either for all pooled or among the two groups following the training period (Table 3). Baseline (pre) values in performance, physiological and technical capacities between-groups are presented in Supplementary Tables.

**Table 2:** Training characteristics (mean ± SD) in 24 endurance transfer athletes during a 6-month XC ski-specific training period.

	**Number (n)**	**Distribution (%)**	
Training days	145 ± 6	83	
Rest days	22 ± 2	13	
Injury/illness days	8 ± 7	4	
**TOTAL**	**Training hours (h)**	**Distribution (%)**	**Number of sessions (n)**
Total training	352 ± 20	100	297 ± 26
**TRAINING FORMS**			
Total training^a^	316 ± 14	90	225 ± 11
XC skiing drills	36 ± 8	10	71 ± 17
**TRAINING FORMS^b^**			
Endurance	264 ± 12	75 (84)	144 ± 9
Strength	38 ± 2	11 (12)	44 ± 2
Speed	14 ± 1	4 (4)	38 ± 2
**INTENSITY DISTRIBUTION^c^**			
LIT	227 ± 10	86	
MIT	13 ± 1	5	
HIT	25 ± 2	9	
**EXERCISE MODE^d^**			
Running	83 ± 5	30	
Skiing skating	109 ± 4	39	
Skiing classic	67 ± 6	24	
Roller-skiing skating	11 ± 1	3	
Roller-skiing classic	8 ± 1	4	

*LIT, low-intensity training; MIT, moderate-intensity training; HIT, high-intensity training*.

a *Total training without including XC skiing drills*.

b *Percent is given as a percentage of total training with and (without XC skiing drills)*.

c *Percent is given as a percentage of total endurance training*.

d *Percent is given as a percentage of total endurance training and speed training*.

**Table 3:** Performance, physiological and technical capacities (mean ± SD) in treadmill roller-ski skating, double-poling ergometry and treadmill running as well as upper-body 1RM strength in 24 endurance transfer athletes during pre-, mid- and post-tests of a 6-month XC ski-specific training period.

	**Pre-test**	**Mid-test**	**Post-test**	**Pre-post**
Body mass (kg)	65.4 ± 9.9	65.4 ± 9.3	65.6 ± 9.5	0.02
**TREADMILL ROLLER-SKI SKATING**				**ES^a^**
V_peak_ (m·s^−1^)	3.85 ± 0.26	4.19 ± 0.35**	4.35 ± 0.37**	1.56
Power V_peak_ (W)	241 ± 45	261 ± 46**	270 ± 47**	1.54
VO_2peak_ (L·min^−1^)	3.93 ± 0.75	4.07 ± 0.73^#^	4.02 ± 0.73	0.12
VO_2peak_ (mL·min^−1^·kg^−1^)	60.0 ± 6.1	62.1 ± 7.1^#^	61.3 ± 7.2	0.19
Maximum respiratory exchange ratio	1.09 ± 0.04	1.09 ± 0.04	1.11 ± 0.05	0.44
Maximum blood lactate (mmol·L^−1^)	8.6 ± 2.1	9.8 ± 1.8	10.0 ± 2.2*	0.65
Peak heart rate (beats·min^−1^)	189 ± 9	192 ± 8**	191 ± 7*	0.24
Peak RPE (1–10)	6.5 ± 1.4	8.5 ± 1.5**	8.7 ± 1.5**	1.41
Submaximal power 4 mmol·L^−1^ (W)	140 ± 36	138 ± 32	165 ± 35**	0.70
Submaximal O_2_-cost (L·min^−1^)	2.86 ± 0.44	2.83 ± 0.41	2.74 ± 0.41*	0.28
Submaximal O_2_-cost (mL·min^−1^·kg^−1^)	43.8 ± 4.0	43.3 ± 3.1	42.0 ± 3.0*	0.51
Submaximal respiratory exchange ratio	0.95 ± 0.05	0.95 ± 0.04	0.92 ± 0.04*	0.22
Submaximal heart rate (beats·min^−1^)	164 ± 12	163 ± 13	157 ± 11*	0.61
Submaximal blood lactate (mmol·L^−1^)	3.4 ± 1.0	3.2 ± 0.9	2.3 ± 0.8**	1.21
Submaximal RPE (1–10)	3.4 ± 0.7	3.3 ± 0.8	2.9 ± 0.8	0.66
Submaximal gross efficiency (%)	12.8 ± 1.1	12.8 ± 1.0	13.4 ± 0.9**	0.59
Submaximal cycle length (m)	5.10 ± 0.40	5.69 ± 0.48**	5.76 ± 0.51**	1.44
Submaximal cycle rate (Hz)	0.49 ± 0.04	0.44 ± 0.04**	0.44 ± 0.04**	1.25
**DOUBLE-POLING ERGOMETRY**				
Power output 5-min performance test (W)	196 ± 43	207 ± 43**	211 ± 45**	0.34
Peak power 5-min performance test (W)	265 ± 59	276 ± 59	273 ± 56	0.14
Power output 30-s Wingate test (W)	332 ± 86	342 ± 84	394 ± 100*	0.66
Peak power output 30-s Wingate test (W)	394 ± 100	434 ± 147	425 ± 127**	0.27
VO_2peak_ (L·min^−1^)	3.76 ± 0.83	3.83 ± 0.78	3.86 ± 0.76	0.12
VO_2peak_ (mL·min^−1^·kg^−1^)	57.3 ± 8.3	58.3 ± 8.5	58.7 ± 7.2	0.18
Maximum respiratory exchange ratio	1.03 ± 0.05	1.05 ± 0.04	1.03 ± 0.04	0.00
Maximum blood lactate (mmol·L^−1^)	12.5 ± 1.9	13.0 ± 2.6	12.7 ± 2.5	0.09
Peak heart rate (beats·min^−1^)	181 ± 10	183 ± 8	181 ± 7	0.00
Peak RPE (1–10)	7.7 ± 1.8	8.4 ± 1.5	8.6 ± 0.9^#^	0.63
**TREADMILL RUNNING**				
V_peak_ (m·s^−1^)	4.05 ± 0.43	4.18 ± 0.37*	4.16 ± 0.35	0.28
VO_2max_ (L·min^−1^)	4.22 ± 0.84	4.34 ± 0.84^#^	4.26 ± 0.76	0.05
VO_2max_ (mL·min^−1^·kg^−1^)	64.4 ± 7.5	66.3 ± 7.4^#^	65.0 ± 7.3	0.08
Maximum respiratory exchange ratio	1.13 ± 0.04	1.15 ± 0.03	1.13 ± 0.04	0.00
Maximum blood lactate (mmol·L^−1^)	10.2 ± 2.7	11.2 ± 1.6	11.4 ± 1.9	0.51
Maximum heart rate (beats·min^−1^)	193 ± 9	194 ± 9	194 ± 8	0.12
Maximum RPE (1–10)	8.0 ± 1.7	8.9 ± 1.2	8.7 ± 1.2	0.47
Submaximal speed 4 mmol·L^−1^ (m·s^−1^)	2.68 ± 0.29	2.57 ± 0.25^#^	2.70 ± 0.28	0.07
**1RM UPPER-BODY STRENGTH**				
Seated pull-down exercise (kg)	57.8 ± 11.1	62.3 ± 12.0**	64.2 ± 11.7**	0.56
Triceps-press exercise (kg)	60.5 ± 11.4	65.4 ± 11.3**	66.6 ± 11.5**	0.53

V_peak_, peak treadmill speed; VO_2peak_, peak oxygen uptake; RPE, rating of perceived exhaustion (1–10); VO_2max_, maximum oxygen uptake. *Significant difference from pre-test (**P < 0.01; ^*^P < 0.05).

# *Tendency toward significant difference from pre-test (P = 0.05–0.1)*.

a *ES of pre-post changes calculated according to Cohens d*.

### Treadmill Roller-Ski Skating

**Figure 1:**
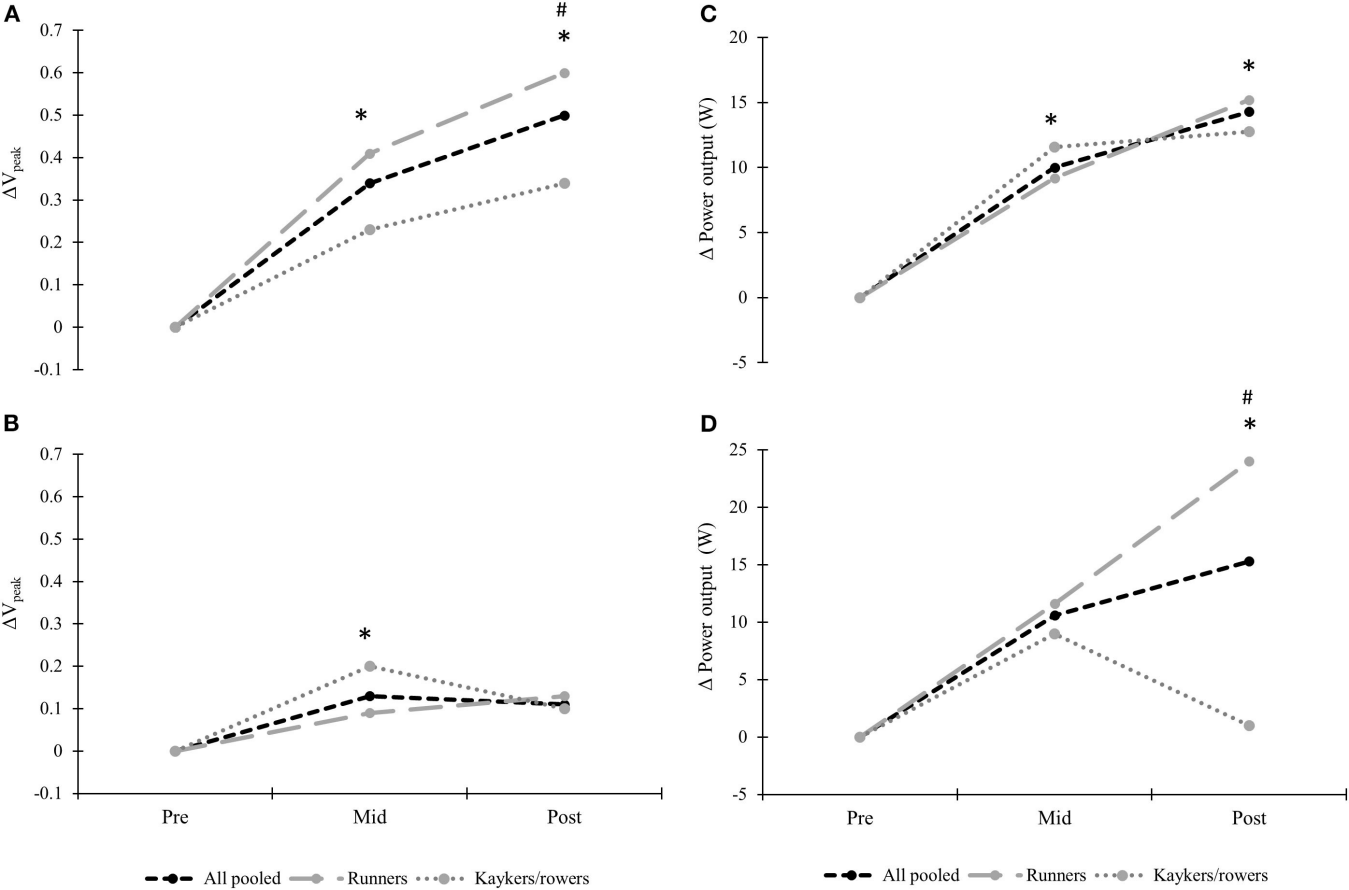
Changes in performance measured as **(A)** peak treadmill speed roller-ski skating, **(B)** peak treadmill speed running, **(C)** average power output during 5-min and **(D)** 30-s double-poling ergometry tests in 24 endurance transfer athletes following a 6-month XC ski-specific training period. *Significant change from pre-test for all pooled (*P* < 0.05). ^#^Significant difference in change from pre-test between-groups (*P* < 0.05).

Performance measured as V_peak_ improved by 9.1 ± 8.0% and 13.1 ± 8.3% for all pooled from pre-mid and pre-post, respectively (both *P* < 0.01; Table 3), with a larger pre-post improvement in runners compared to kayakers/rowers (15.7 ± 8.8%, vs. 8.9 ± 5.2%; *P* < 0.01; Figure 1A). There was a tendency toward improved absolute and body mass normalized VO_2peak_ (3.8 ± 6.0% and 3.6 ± 5.7%; both *P* = 0.08–0.09) from pre-mid for all pooled, but no significant changes were found comparing pre-post. However, runners improved both absolute and body mass normalized VO_2peak_ (4.9 ± 7.0% and 4.0 ± 7.1%; *P* < 0.05) from pre-post which differed from a corresponding non-change in kayakers/rowers (*P* < 0.05). Skating-VO_2peak_ reached on average 94.2 ± 6.1% of running-VO_2max_ at pre-testing for all pooled and did not change following the 6-month training period. No between group differences in skating-VO_2peak_/VO_2max_ ratios were found.

**Figure 2:**
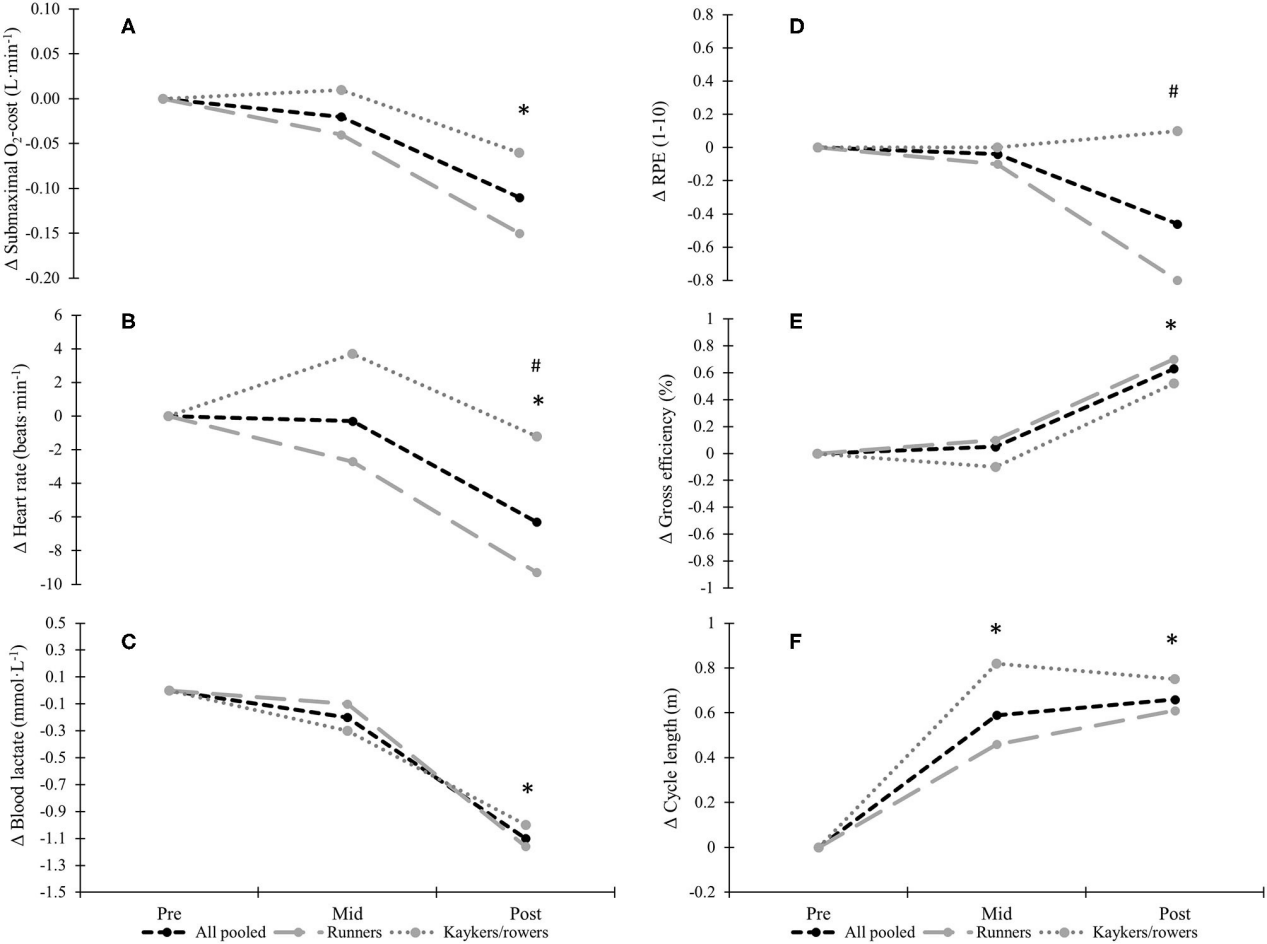
Changes in **(A)** O_2_-cost, **(B)** heart rate, **(C)** blood lactate values, **(D)** rating of perceived exertion, **(E)** gross efficiency and **(F)** cycle length during submaximal treadmill roller-ski skating in 24 endurance transfer athletes following a 6-month XC ski-specific training period. *Significant change from pre-test for all pooled (*P* < 0.05). ^#^Significant difference in change from pre-test between-groups (*P* < 0.05).

No significant changes were found from pre-mid during submaximal roller-ski skating, whereas pre-post changes revealed significantly lower physiological cost using the same absolute speed, with a −1.9 ± 2.6 mL·min^−1^·kg^−1^, −6 ± 10 beats·min^−1^, −0.03 ± 0.05, and −0.9 ± 0.7 mmol·L^−1^ reduction in O_2_-cost, heart rate, respiratory exchange ratio (RER), and blood lactate values, respectively, for all pooled (all *P* < 0.05; Table 3; Figures 2A–C). Significantly larger reduction in heart rate was found from pre-post in runners compared to kayakers/rowers (−9 ± 11 vs. −1 ± 6 beats·min^−1^; *P* < 0.05; Figure 2B). Moreover, RPE using the same absolute speed was unchanged for all pooled but was significantly reduced by−0.8 ± 0.8 points in runners comparing pre-post (*P* < 0.01), being significantly different from a non-change in kayakers/rowers (*P* < 0.01; Figure 2D). Power at 4 mmol·L^−1^ improved significantly from pre-post for all pooled and runners (21.3 ± 24.6% and 28.2 ± 26.3%; both *P* < 0.05) which differed from a non-significant improvement in kayakers/rowers (*P* < 0.05). Furthermore, GE improved by 0.6 ± 0.6% points for all pooled comparing pre-post changes (both *P* < 0.05; Figure 2E), with no significant between-group differences in change observed.

Submaximal roller-ski skating at same absolute speed revealed improvements of 11.9 ± 8.7% and 13.1 ± 7.1% in cycle length from pre-mid and pre-post, respectively (Both *P* < 0.01; Figure 2F). The increased cycle lengths were accompanied by −10.1 ± 6.9% and −11.3 ± 5.6% reductions in cycle rate from pre-mid and pre-post, respectively (both *P* < 0.01). No significant differences in change of cycle characteristics were found between-groups.

### Ergometer Double-Poling

Performance measured as average power output in the 5-min performance test improved by 5.4 ± 5.3% and 7.8 ± 9.3% from pre-mid and pre-post, respectively (both *P* < 0.01; Table 3, Figure 1C), with no between-group differences in change observed. During the 30-s Wingate test, no significant changes were found pre-mid, but pre-post revealed significant improvements in average power output for all pooled and runners (5.0 ± 6.2% and 7.6 ± 6.3%; both *P* < 0.05), being significantly different from a small reduction in power output among kayakers/rowers (*P* < 0.05; Figure 1D). Lastly, no significant changes in VO_2peak_ in the 5-min performance test were observed for all pooled following the training period, which did not differ between the two groups. Double-poling-VO_2peak_ reached on average 89.4 ± 10.2% of running-VO_2max_ for all pooled at pre-testing, with no significant changes neither for all pooled nor between-groups during the training period.

### Treadmill Running

Changes in physiological capacities and performance during treadmill running for all pooled are presented in Table 3 and Figure 1B. V_peak_ improved from pre-mid (3.5 ± 4.7%; *P* < 0.05) but no significant changes were found from pre-post which did not differ between the two groups (Figure 1B). There was a tendency toward improved absolute and body mass normalized VO_2max_ from pre-mid for all pooled (3.4 ± 3.9% and 3.1 ± 4.1%; *P* = 0.08–0.09), whereas no significant improvements in running VO_2max_ were observed from pre-post, with no differences between runners and kayakers/rowers. Speed at 4 mmol·L^−1^ tended to decrease by −3.9 ± 4.9% for all pooled from pre-mid (*P* < 0.05), whilst pre-post revealed no significant changes in speed at 4 mmol·L^−1^ with no differences in change between-groups.

### 1RM Upper-Body Strength

Changes in 1RM upper-body strength for all pooled are presented in Table 3. 1RM seated pull-down strength improved by 8.2 ± 9.3% and 11.7 ± 8.8% from pre-mid and pre-post, respectively (*P* < 0.01), with no significant differences in change observed between-groups. 1RM strength in the triceps press showed significant improvements of 8.8 ± 7.3% and 10.7 ± 8.7% from pre-mid and pre-post for all pooled (both *P* < 0.01) with no differences between runners and kayakers/rowers.

In all the above cases, ES of pre-post changes for all athletes pooled are presented in Table 3, whereas ES of pre-post changes between-groups are presented in Supplementary Tables 1, 2.

## Discussion

The present study examined the development of performance, physiological and technical capacities as well as the effect of sport background among runners and kayakers/rowers when transferred to XC skiing over a 6-month training period. The main findings were as follows: (1) Performance in roller-ski skating improved by 13%, with a larger improvement in runners compared to kayakers/rowers (16% vs. 9%), whereas running performance did not change; (2) Average power output during 5-min and 30-s ergometer double-poling tests improved by 8% and 5%, with improvement on the 30-s test revealed only among runners (8% vs. −2% in kayakers/rowers); (3) VO_2max_ in running and VO_2peak_ double-poling did not change either in all athletes pooled or in any of the groups, whereas VO_2peak_ in treadmill roller-ski skating improved 5% in runners that was significantly greater than the unchanged values in kayakers/rowers. These results were further supported by the strengths of the ES presented in the Supplementary Tables.

### General vs. Sport-Specific Capacities

Although some scientific evidence of individuals (Rønnestad et al., 2019) and groups (Bullock et al., 2009; MacNamara and Collins, 2015) who have been transferred between sports on the elite level exist, this is the first systematic documentation of this process in the scientific literature. Here, we showed large improvements in sport-specific performance indicators (roller-ski skating and double-poling ergometry) following a 6-month XC ski-specific training period in a group of endurance athletes transferring to XC skiing. This was coincided by concurrent improvements in roller-ski gross efficiency and cycle length, and by upper-body 1RM strength. In contrast, running performance and VO_2max_ remained unchanged. These findings were as expected due to the combined introduction of dedicated sport-specific training and the already well-developed endurance capacities in these athletes.

Here, we provide a framework for long-term monitoring of training and sport-specific performance-development in athletes transferring between sports. The degree of improvement in the different exercise modes found seems associated with the technical complexity of modes, where roller-ski skating shows the largest improvements due to more sport-specific and complex technical requirements compared to ergometer double-poling and running. The revealed magnitude of development in key sport-specific capacities clearly supports that longitudinal monitoring of sport-specific laboratory capacities is important in both talent transfer athletes and XC skiers in general (Sandbakk et al., 2011b, 2016a). In addition, understanding the differences between the performance and physiological development in general vs. sport-specific modes might be particularly important in the process of transferring athletes between sports that requires different technical demands. However, it needs to be established to what extent the development of different capacities in both general and sport-specific modes continue over longer time-spans, both in talent transfer athletes and in XC skiers.

Aerobic energy delivery capacities (i.e., VO_2peak_ and VO_2max_) did not change significantly in either of exercise modes after 6-months of XC ski-specific training. This was not unexpected in running among these already endurance-trained athletes, but unexpected in more sport-specific modes as roller-ski skating and double-poling ergometry. However, surprisingly high VO_2peak_/VO_2max_ ratios already at baseline were found in roller-ski skating and double-poling ergometry in these unexperienced XC skiers, with ratios close to those reported among elite XC skiers (Sandbakk and Holmberg, 2017; Losnegard, 2019). This could indicate a limited potential for further improving this VO_2peak_/VO_2max_ ratio, combined with test protocols allowing the athletes to reach relatively high maximum physiological values without being too much limited by their technical inexperience. Nevertheless, this indicates that the improvement in performance during roller-ski skating and double-poling must be explained by development of other capacities such as improved technique (i.e., longer cycle length) and efficiency, as found in the current study. Another possible mechanism might be that the athletes improved other factors than those measured in our design, which have contributed to explain parts the observed improvements in sport-specific performance. This could be factors such as higher fractional utilization of VO_2peak_ and/or improved anaerobic energy delivery capacity (e.g., blood buffering capacity; Losnegard et al., 2012, 2013).

In this context, the athletes showed improved technique-specific upper-body strength that probably enhanced their capacity to produce longer cycle lengths and higher speeds independent of changes in VO_2peak_. Such improvements in technique-specific propulsion have previously been associated with longer cycle lengths and better work economy/efficiency in XC skiers (Sandbakk et al., 2010; Sandbakk and Holmberg, 2017). In roller-ski skating, the largest improvement in cycle lengths were found from pre-mid, which corresponds well with the largest improvement in performance (i.e., peak treadmill speed). This further supports that improved technique and efficiency are main explanatory factors for improved performance in roller-ski skating among this group of athletes. Furthermore, large improvements were found in 1RM upper-body strength in exercises previously proven relevant for performance in XC skiing (Losnegard et al., 2011). Overall, this suggests that the development of performance during both roller-ski skating and double-poling is coupled with improved strength/power, leading to better sport-specific propulsion.

Following the improved skiing efficiency that occurred to the greatest extent from mid-post, lower physiological and perceptual costs (i.e., heart rate, RER, blood lactate values, and RPE) were found when athletes were compared at the same absolute submaximal speed and power outputs of 4 mmol·L^−1^ increased. This indicates that the submaximal stages were less demanding and that sport-specific indices of anerobic threshold while treadmill roller-ski skating were improved following the training period. The reason for these submaximal adaptations occurring at a later timepoint compared to maximal capacities (i.e., performance) is not known but could imply that a longer timeframe is required to develop skiing efficiency and subsequently induce these adaptations. Finally, the athletes showed significantly higher RPE, heart rate, and blood lactate values during the sport-specific maximal tests at post-testing, most likely brought about by improved ability to push themselves and increase effort in a new exercise mode.

### Runners vs. Kayakers/Rowers

Runners improved roller-ski skating performance and power output at 4 mmol·L^−1^ to a greater extent than kayakers/rowers from pre-post, accompanied by a 5% increase in VO_2peak_ that was larger than the unchanged values for kayakers/rowers. In addition, runners demonstrated a tendency toward improved VO_2max_ in running (moderate ES), meaning that runners show greater endurance adaptations following 6-months of XC ski-specific training. However, no group differences in the development of cycle length, skiing efficiency, or 1RM upper-body strength were revealed. This could possibly imply that despite similar potential for developing technical and strength/power capacities in both groups, runners are more likely to respond physiologically positive to high loads of endurance training and specifically to induce adaptations that improve aerobic energy delivery capacity. However, the underlying mechanisms for these findings, as well as the individual variations in training response following periods of standardized XC ski-specific training, are not known and require further examination.

There was clearly a progressive improvement in runners during the entire 6-month training period, indicated by large developments in sport-specific capacities in addition to a tendency toward improved capacities in running (Supplementary Table 1). In contrast, plateaus or decreases in the development of these capacities were found from mid-post among kayakers/rowers. The reason for these different findings across groups is not known, but they imply a generally lower training response in kayakers/rowers. This might be explained by a lower tolerance for high loads of endurance training in a new exercise mode in this group of athletes, possibly leading to negative training outcomes in some of the athletes (e.g., maladaptation, non-functional overreaching and overtraining). In particular, runners might have been more familiar with the main training modes being utilized since a large amount of running was performed (30% of all endurance training), and classical skiing involves movements more closely related to the movement patterns of their sports background. In addition, slightly higher incidents of injury and illness were reported in the group of kayakers/rowers with most of these days being reported during the last 3-months (mid-post) of the training period, which supports the theory of negative training outcomes in some of the kayakers/rowers, leading to the observed plateaus and lack of progress from mid-post. A potential mechanism explaining increased incidents of injury among kayakers/rowers might be their ~6 kg higher body mass, which are likely of relevance considering the amounts of running performed. Overall, this led to a lower training compliance and a tendency for lower amount of training performed among kayakers/rowers. Although this difference in total training volume was only minor (5%), it could potentially have elicited a greater overall training stimulus and thus contributed to better adaptations and a larger sport-specific development in the group of runners. A potential limitation of our design was the relatively large variation in the duration of introduction period to roller-skiing before the pre-tests. However, we found no difference in the duration of the introduction period between kayakers/rowers and runners or significant correlations between the duration of the introduction period and pre-post test changes within groups or with all athletes pooled.

Although power output during double-poling ergometry and 1RM upper-body strength did not differ significantly between-groups at baseline, these values tended to be higher among kayakers/rowers. Therefore, a larger development of power output on the 30-s double-poling ergometry test in runners was as expected due to their larger potential to develop upper-body strength and power capacities compared to kayakers/rowers which largely depends on upper-body propulsion. However, contrary to this hypothesis, no differences in the development of upper-body 1RM strength were found between-groups. Therefore, the larger improvement in runners, must be explained by factors not examined here.

## Conclusion

Transfer of individual athletes and groups to new sports are commonly used in sports practice although the knowledge concerning such transitions has largely been based on anecdotal evidence. By systematically documenting the effects of talent transfer to XC skiing, we provide novel understanding of the development of performance, physiological and technical capacities among endurance athletes transferring from running, rowing, or kayaking. Specifically, we found large improvements in sport-specific performance (i.e., roller-ski skating and double-poling) following 6-months of XC ski-specific training. This was associated with enhanced skiing efficiency, longer cycle lengths, and improved 1RM upper-body strength while no changes occurred in the maximal aerobic energy delivery capacities (i.e., VO_2max_ or VO_2peak_). In addition, larger sport-specific development was found in athletes transferring from running compared to kayaking or rowing. Accordingly, follow-up studies should aim to further explain the factors underlying the differences in sports background as well as the long-term effects of transferring athletes between sports at the elite level.

## Data Availability Statement

The raw data supporting the conclusions of this article will be made available by the authors, without undue reservation.

## Ethics Statement

The studies involving human participants were reviewed and approved by Norwegian Center for Research Data. The patients/participants provided their written informed consent to participate in this study.

## Author Contributions

RT, T-AH, and ØS designed the study. RT, T-AH, and XC performed the data collection. RT performed the data and statistical analysis: RT, T-AH, and ØS contributed to the interpretation of the results. RT and ØS wrote the draft manuscript. RT, T-AH, XC, and ØS contributed to the final manuscript. All authors contributed to the article and approved the submitted version.

## Conflict of Interest

The authors declare that the research was conducted in the absence of any commercial or financial relationships that could be construed as a potential conflict of interest.
