# Supplementary material for: Development of Performance, Physiological and Technical Capacities During a Six-Month Cross-Country Skiing Talent Transfer Program in Endurance Athletes
Source: Front Sports Act Living. 2020 Aug 12;2:103. doi: 10.3389/fspor.2020.00103 (PMC7739832; doi:10.3389/fspor.2020.00103)
Supplement: Supplementary file 1 [file Table_1.docx]

| **SUPPLEMENTARY TABLE 1.** Performance, physiological and technical capacities (mean ± SD) in treadmill running and treadmill roller-ski skating in runners (n=15) and kayakers/rowers (n=9) participating in an athlete-transfer program during pre-, mid- and post-tests of a six-month XC ski-specific training period. | | | | | | | |
| --- | --- | --- | --- | --- | --- | --- | --- |
|  | **Pre-test** | | **Mid-test** | | **Post-test** | | **Pre-post** |
|  | **Running** | **Kayaking/rowing** | **Running** | **Kayaking/rowing** | **Running** | **Kayaking/rowing** | **ES^a^** |
| Body mass (kg) | 62.1 ± 8.6 | 71.0 ± 9.3† | 62.4 ± 8.7 | 70.5 ± 8.1 | 62.7 ± 8.7 | 70.4 ± 8.8 | 0.20 |
| **Treadmill roller-ski skating** |  |  |  |  |  |  |  |
| V_peak_ (m·s^-1^) | 3.85 ± 0.27 | 3.83 ± 0.24 | 4.26 ± 0.37** | 4.07 ± 0.27** | 4.45 ± 0.38** | 4.17 ± 0.30**# | 1.02 |
| Power V_peak_ (W) | 229 ± 40 | 261 ± 47 | 252 ± 42** | 274 ± 49** | 265 ± 46** | 278 ± 47**# | 1.15 |
| VO_2peak_ (L·min^-1^) | 3.87 ± 0.71 | 4.03 ± 0.81 | 4.07 ± 0.74* | 4.06 ± 0.73 | 4.05 ± 0.73* | 3.97 ± 0.72# | 0.94 |
| VO_2peak_ (mL·min^-1^·kg^-1^) | 62.1 ± 5.2 | 56.4 ± 5.7† | 65.0 ± 6.9* | 57.3 ± 4.4 | 64.5 ± 6.7* | 56.0 ± 4.1# | 0.68 |
| Maximum respiratory exchange ratio | 1.09 ± 0.05 | 1.09 ± 0.03 | 1.09 ± 0.05 | 1.10 ± 0.02 | 1.10 ± 0.04 | 1.11 ± 0.05 | 0.44 |
| Maximum blood lactate (mmol·L^-1^) | 8.3 ± 2.1 | 9.0 ± 2.1 | 10.1 ± 1.9 | 9.4 ± 1.3 | 9.9 ± 2.1* | 10.4 ± 2.2* | 0.11 |
| Peak heart rate (beats·min^-1^) | 191 ± 9 | 184 ± 7† | 194 ± 8** | 188 ± 7** | 193 ± 7* | 188 ± 6.7* | 0.58 |
| Peak RPE (1-10) | 6.2 ± 1.4 | 6.7 ± 1.2 | 7.8 ± 1.5** | 8.4 ± 1.4** | 8.5 ± 1.1** | 8.9 ± 0.9** | 0.18 |
| Submaximal power 4 mmol·L^-1^ (W) | 127 ± 29 | 160 ± 35† | 130 ± 32 | 151 ± 30 | 160 ± 33** | 173 ± 36# | 1.15 |
| Submaximal O_2_-cost (L·min^-1^) | 2.82 ± 0.45 | 2.92 ± 0.43 | 2.79 ± 0.44 | 2.90 ± 0.44 | 2.71 ± 0.39* | 2.81 ± 0.43* | 0.06 |
| Submaximal O_2_-cost (mL·min^-1^·kg^-1^) | 45.4 ± 3.9 | 41.0 ± 2.4† | 44.7 ± 2.8 | 41.1 ± 2.0 | 43.2 ± 2.9* | 38.8 ± 1.7* | 0.37 |
| Submaximal respiratory exchange ratio | 0.95 ± 0.05 | 0.96 ± 0.04 | 0.96 ± 0.04 | 0.94 ± 0.03 | 0.92 ± 0.04* | 0.93 ± 0.05* | 0.00 |
| Submaximal heart rate (beats·min^-1^) | 168 ± 12 | 156 ± 10† | 165 ± 11 | 160 ± 14 | 159 ± 13* | 155 ± 6# | 0.91 |
| Submaximal blood lactate (mmol·L^-1^) | 3.5 ± 0.8 | 3.1 ± 1.2 | 3.4 ± 0.9 | 2.8 ± 0.8 | 2.4 ± 0.8** | 2.1 ± 0.6** | 0.14 |
| Submaximal RPE (1-10) | 3.4 ± 0.8 | 3.3 ± 0.5 | 3.3 ± 0.9 | 3.3 ± 0.5 | 2.6 ± 0.7** | 3.4 ± 0.7# | 1.12 |
| Submaximal gross efficiency (%) | 12.4 ± 1.1 | 13.4 ± 0.7† | 12.5 ± 0.8 | 13.4 ± 0.9 | 13.1 ± 0.9** | 14.0 ± 0.6** | 0.25 |
| Submaximal cycle length (m) | 5.13 ± 0.44 | 5.05 ± 0.32 | 5.59 ± 0.56** | 5.87 ± 0.32** | 5.74 ± 0.56** | 5.80 ± 0.40** | 0.40 |
| Submaximal cycle rate (Hz) | 0.49 ± 0.04 | 0.50 ± 0.03 | 0.45 ± 0.05** | 0.43 ± 0.02** | 0.44 ± 0.04** | 0.43 ± 0.03** | 0.33 |
| **Treadmill running** |  |  |  |  |  |  |  |
| V_peak_ (m·s^-1^) | 4.22 ± 0.40 | 3.77 ± 0.33† | 4.31 ± 0.38* | 3.97 ± 0.25* | 4.32 ± 0.33 | 3.90 ± 0.16 | 0.12 |
| VO_2max_ (L·min^-1^) | 4.23 ± 0.80 | 4.20 ± 0.91 | 4.35 ± 0.81 | 4.34 ± 0.88# | 4.35 ± 0.78 | 4.10 ± 0.69 | 0.84 |
| VO_2max_ (mL·min^-1^·kg^-1^) | 67.8 ± 6.3 | 58.6 ± 5.5† | 69.4 ± 6.5 | 61.0 ± 5.7# | 69.2 ± 5.7 | 58.0 ± 3.0 | 0.56 |
| Maximum respiratory exchange ratio | 1.14 ± 0.03 | 1.12 ± 0.04 | 1.14 ± 0.03 | 1.16 ± 0.03 | 1.13 ± 0.05 | 1.12 ± 0.04 | 0.28 |
| Maximum blood lactate (mmol·L^-1^) | 10.4 ± 3.2 | 9.9 ± 1.4 | 10.8 ± 1.9 | 11.7 ± 0.9 | 11.8 ± 2.0 | 10.8 ± 1.5 | 0.18 |
| Maximum heart rate (beats·min^-1^) | 195 ± 9 | 190 ± 11 | 195 ± 10 | 192 ± 7 | 196 ± 8 | 191 ± 8 | 0.02 |
| Maximum RPE (1-10) | 7.7 ± 1.7 | 8.7 ± 1.3 | 9.0 ± 1.2 | 8.8 ± 0.8 | 8.5 ± 1.4 | 9.0 ± 0.7 | 0.30 |
| V_peak_, peak treadmill speed; VO_2max_, maximum oxygen uptake; RPE, rating of perceived exhaustion (1-10); VO_2pea_, peak oxygen uptake. †Significant difference between groups at baseline (pre). *Significant change from pre-test (P<0.05). # Significant difference in change from pre-test between-groups (P<0.05). ^a^ES of pre-post change between-groups calculated according to Cohens d. | | | | | | | |
