# Supplementary material for: Development of Performance, Physiological and Technical Capacities During a Six-Month Cross-Country Skiing Talent Transfer Program in Endurance Athletes
Source: Front Sports Act Living. 2020 Aug 12;2:103. doi: 10.3389/fspor.2020.00103 (PMC7739832; doi:10.3389/fspor.2020.00103)
Supplement: Supplementary file 2 [file Table_2.docx]

| **SUPPLEMENTARY TABLE 2.** Performance and physiological capacities during double-poling ergometry as well as upper-body 1RM strength (mean ± SD) in runners (n=15) and kayakers/rowers (n=9) participating in an athlete-transfer program during pre-, mid- and post-test of a six-month XC ski-specific training period. | | | | | | |  |
| --- | --- | --- | --- | --- | --- | --- | --- |
|  | **Pre-test** | | **Mid-test** | | **Post-test** | | **Pre-post** |
|  | **Running** | **Kayaking/rowing** | **Running** | **Kayaking/rowing** | **Running** | **Kayaking/rowing** | **ES^a^** |
| **Double-poling ergometry** |  |  |  |  |  |  |  |
| Power output 5-min performance test (W) | 188 ± 43 | 210 ± 40 | 198 ± 43** | 221 ± 40** | 204 ± 44** | 222 ± 43** | 0.15 |
| Peak power output 5-min performance test (W) | 255 ± 61 | 282 ± 53 | 261 ± 60 | 300 ± 50 | 265 ± 62 | 285 ± 42 | 0.20 |
| Power output 30-sec Wingate test (W) | 316 ± 72 | 358 ± 99 | 328 ± 78 | 376 ± 88 | 340 ± 79** | 350 ± 94# | 1.38 |
| Peak power output 30-sec Wingate test (W) | 375 ± 80 | 425 ± 122 | 409 ± 123 | 475 ± 171 | 407 ± 95** | 455 ± 162** | 0.02 |
| VO_2peak_ (L·min^-1^) | 3.76 ± 0.86 | 3.76 ± 0.79 | 3.86 ± 0.77 | 3.78 ± 0.79 | 3.88 ± 0.73 | 3.82 ± 0.80 | 0.44 |
| VO_2peak_ (mL·min^-1^·kg^-1^) | 60.1 ± 8.8 | 52.6 ± 4.4† | 61.5 ± 8.3 | 53.2 ± 5.6 | 61.7 ± 6.7 | 53.8 ± 4.8 | 0.14 |
| Maximum respiratory exchange ratio | 1.04 ± 0.04 | 1.00 ± 0.05 | 1.05 ± 0.03 | 1.04 ± 0.05 | 1.04 ± 0.03 | 1.02 ± 0.04 | 0.63 |
| Maximum blood lactate (mmol·L^-1^) | 12.6 ± 2.0 | 12.2 ± 1.6 | 12.2 ± 2.6 | 14.2 ± 2.0 | 12.8 ± 2.9 | 13.8 ± 1.2 | 0.10 |
| Peak heart rate (beats·min^-1^) | 184 ± 11 | 177 ± 6 | 185 ± 9 | 180 ± 6 | 183 ± 8 | 178 ± 5 | 0.51 |
| Peak RPE (1-10) | 7.2 ± 2.1 | 8.6 ± 0.7† | 8.1 ± 1.7 | 8.8 ± 1.2 | 8.5 ± 1.0 | 8.8 ± 0.8 | 0.81 |
| **1RM upper-body strength** |  |  |  |  |  |  |  |
| Seated pull-down exercise (kg) | 54.7 ± 8.7 | 62.9 ± 12.5 | 58.6 ± 10.2* | 66.8 ± 13.2* | 61.3 ± 10.7** | 69.0 ± 11.8** | 0.13 |
| Triceps-press exercise (kg) | 57.7 ± 10.5 | 65.2 ± 11.4 | 63.3 ± 10.4** | 69.0 ± 11.8** | 64.5 ± 10.7** | 70.1 ± 11.8** | 0.44 |
| VO_2peak_, peak oxygen uptake; RPE, rating of perceived exhaustion (1-10). †Significant difference between-groups at baseline (pre). *Significant change from pre-test (P<0.05). # Significant difference in change from pre-test between-groups (P<0.05). ^a^ES of pre-post change between-groups calculated according to Cohens d. | | | | | | | |
